# An investigation of reasoning by analogy in schizophrenia and autism spectrum disorder

**DOI:** 10.3389/fnhum.2014.00517

**Published:** 2014-08-20

**Authors:** Daniel C. Krawczyk, Michelle R. Kandalaft, Nyaz Didehbani, Tandra T. Allen, M. Michelle McClelland, Carol A. Tamminga, Sandra B. Chapman

**Affiliations:** ^1^Center for BrainHealth^®^, School of Behavioral and Brain Sciences, The University of Texas at DallasDallas, TX, USA; ^2^Department of Psychiatry, University of Texas Southwestern Medical CenterDallas, TX, USA

**Keywords:** analogy, reasoning, schizophrenia, autism spectrum disorder, distraction

## Abstract

Relational reasoning ability relies upon by both cognitive and social factors. We compared analogical reasoning performance in healthy controls (HC) to performance in individuals with Autism Spectrum Disorder (ASD), and individuals with schizophrenia (SZ). The experimental task required participants to find correspondences between drawings of scenes. Participants were asked to infer which item within one scene best matched a relational item within the second scene. We varied relational complexity, presence of distraction, and type of objects in the analogies (living or non-living items). We hypothesized that the cognitive differences present in SZ would reduce relational inferences relative to ASD and HC. We also hypothesized that both SZ and ASD would show lower performance on living item problems relative to HC due to lower social function scores. Overall accuracy was higher for HC relative to SZ, consistent with prior research. Across groups, higher relational complexity reduced analogical responding, as did the presence of non-living items. Separate group analyses revealed that the ASD group was less accurate at making relational inferences in problems that involved mainly non-living items and when distractors were present. The SZ group showed differences in problem type similar to the ASD group. Additionally, we found significant correlations between social cognitive ability and analogical reasoning, particularly for the SZ group. These results indicate that differences in cognitive and social abilities impact the ability to infer analogical correspondences along with numbers of relational elements and types of objects present in the problems.

## Introduction

Reasoning ability is a fundamental aspect of cognition. Humans are capable of drawing together diverse information in order to generate new inferences and discoveries (Penn et al., 2008; Krawczyk, 2012). An important skill driving reasoning and inference is the ability to consider the relations among objects, rather than the perceptual and sensory aspects of the objects themselves (Gentner, 1983; Hummel and Holyoak, 1997, 2003). Relational reasoning ability develops with age and increases as relational knowledge is acquired in childhood (Gentner and Toupin, 1986; Goswami and Brown, 1989; Goswami, 2001) and remains robust throughout the adult lifespan (Goswami and Brown, 1989; Viskontas et al., 2004). Developmental studies of analogical reasoning have shown consistent increases in relational matching with objects (Halford et al., 1994), words (Gentner and Rattermann, 1991), and visual scenes (Markman and Gentner, 1993; Richland et al., 2006) as children age. Cognitive disorders reduce relational reasoning abilities. In the current study, we evaluated three aspects of scene analogical reasoning in healthy individuals and in individuals with either Autism Spectrum Disorder (ASD) or schizophrenia (SZ), both groups exhibiting cognitive and social impairments. Such comparisons may jointly illuminate important processes in analogical reasoning and clarify how reasoning is affected by social cognitive disorders.

Analogical reasoning involves several cognitive processes including perception, semantic memory, and working memory (see Krawczyk, 2012 for review). Prior studies of scene analogy indicate that working memory and executive functions are particularly important. Relational matching in scene analogy tasks is reduced by concurrent working memory load (Waltz et al., 2000), as well as under stressful and anxiety provoking conditions that reduce working memory (Tohill and Holyoak, 2000; Vendetti et al., 2012). Further, in developmental studies relational matching has been shown to emerge around age six and continuing to develop with age and increases in semantic knowledge and working memory (Gentner and Toupin, 1986; Goswami and Brown, 1989; Goswami, 2001). One of the reasons working memory is important in analogical reasoning is that sometimes analogs have multiple relations. For example a cat can be chasing a mouse, while also being chased by a dog. In a similar situation, a girl may be chasing her pet, while her brother chases her. Keeping in mind which individuals occupy agent and patient roles in these two scenarios can tax working memory load. Such problems are often more difficult to solve and require greater processing time (Cho et al., 2007; Krawczyk et al., 2010). Cho et al. (2007) reported that requiring maintenance of information relevant to solving analogies over longer durations similarly decreases reasoning performance. Working memory interfaces with semantic memory in relational reasoning, as categorizing incoming information and noticing relationships among objects and the rules by which they interact are both critically important (Kotovsky and Gentner, 1996; Krawczyk et al., 2005). Scene analogy performance is a useful measure of cognition in disorders, because of this emphasis on working memory and semantic memory. Such tasks may have greater ecological validity than more traditional neuropsychological measures that focus on one construct alone. In the current study we combined a scene analogy task with neuropsychological measures of cognitive and social function in order to elucidate the relationships between these abilities in ASD and SZ.

Prior patient studies of relational reasoning have focused on the role of executive function abilities, including working memory. Individuals with Frontotemporal Lobar Degeneration (FTLD) with an onset within the prefrontal cortex show reduced levels of relational responding on scene analogies (Morrison et al., 2004) and four-term analogies (Krawczyk et al., 2008) along with other forms of relational reasoning (Waltz et al., 1999; Goel et al., 2009). Traumatic Brain Injuries (TBI) can also lead to impairments in executive functioning (see Levin and Hanten, 2005 for a review). Krawczyk et al. (2010) reported that adolescents TBI also show reductions in scene analogy performance. Frontal executive disruption often leads to a greater tendency to select non-relational distractor items that match on the basis of perceptual similarity only (Morrison et al., 2004; Krawczyk et al., 2008, 2010). Such matches are also characteristic of younger children who have not yet fully exhibited a shift toward relational responding (Goswami and Brown, 1989; Goswami, 2001). Executive functions important for relational reasoning appear to be both managing relational complexity in working memory (Halford et al., 1994) and maintaining inhibitory control over the task so that decisions are not made based on semantic or perceptual similarity (Krawczyk et al., 2010). In addition to executive function, social cognitive processing is likely an important target for additional research.

Individuals with mild ASD present with social deficits, but may not show evidence of language delays (Bowman, 1988). Reasoning ability in ASD exhibits a mixed profile necessitating further investigation. Individuals with mild ASD symptoms may perform at the level of neurotypical individuals on abstract reasoning ability, as measured by visuo-spatial intelligence tests (Hayashi et al., 2008) and show typical levels of understanding pragmatics in language. Reasoning abilities may not be constrained by verbal intelligence, especially at the mild end of the Autism spectrum (Pijnacker et al., 2009a). Meanwhile, individuals with ASD have deficits in reasoning about others states of mind, or mentalizing (Blackshaw et al., 2001), as well as certain aspects of pragmatic reasoning (Pijnacker et al., 2009a,b). Morsanyi and Holyoak (2010) investigated analogy performance using a scene analogical reasoning task and reported that children with ASD (excluding cases of pervasive developmental delay) were unimpaired on scene analogies despite social deficits. These results suggest intact ability to handle greater relational complexity in analogical reasoning in ASD. Sahyoun et al. (2009) compared four-term (A:B::C:D) picture analogy performance in individuals with more severe ASD, less severe ASD and neurotypical individuals. The less-severe ASD group showed similar strong performance to controls in visual and semantic analogical reasoning and the autism group showed only longer response times. Despite these strong visuo-spatial and relational reasoning skills in ASD, there may be variations in reasoning with socially-relevant relational content. To date there have been no studies of the influence of social context in relational reasoning, despite ASD symptomatology that indicates deficits in certain areas of inference, as well as mentalizing deficits. It is important to note that ASD encompasses a range of symptoms with a widely heterogeneous severity level (Lai et al., 2014). We focus in the current manuscript on mild ASD lacking significant language delays.

Another relevant comparison group for examining reasoning differences is SZ, which is associated with impairments in several cognitive domains, including executive function, declarative memory, working memory and processing speed (Heinrichs and Zakzanis, 1998; Green et al., 2000; Dickinson et al., 2004; Nuechterlein et al., 2004; Tamminga et al., 2010). There is a broad literature indicating delusional thinking in SZ, which is considered to be a major deficit of reasoning (Startup et al., 2008; Langdon et al., 2010), but there are relatively few laboratory-based studies of reasoning in SZ. Evidence suggests that individuals with SZ may be impaired when performing analogy problems, which may be clinically useful to examine. Individuals with SZ also exhibit basic executive function deficits on tasks that involve working memory and cognitive control (Elliott et al., 1995; Matheson and Langdon, 2008; Snyder et al., 2008), mental functions that form the foundation of abstract reasoning abilities (Holyoak and Kroger, 1995; Krawczyk, 2012). There is also abundant evidence that individuals with SZ exhibit dysfunctions in mentalizing abilities (Frith and Corcoran, 1996; Doody et al., 1998; Ziv et al., 2011), and emotion recognition (Pinkham et al., 2008)—abilities that are important for social cognition. The rather limited analogical reasoning evidence reported in the literature indicates that individuals with SZ exhibit deficits in forming analogical correspondences between stories using a task developed by Gick and Holyoak (1983) (Simpson and Done, 2004). Thus, SZ is likely to impair scene analogy performance due to both cognitive control and social cognitive deficits.

In the current experiment, we tested the effects of cognitive and social deficits on analogical reasoning across ASD, SZ, and healthy controls (HC) using both a scene analogy task and several social and cognitive tests in a neuropsychological battery. The analogy experiment required inferences based on either relational correspondences, or perceptual similarities. We used a task adapted from Richland et al. (2006) which requires participants to compare two line drawn pictures consisting of three to four key objects that match based on the relations (e.g., chasing, or reaching) present in the two pictures. Relational complexity is varied including either one or two relations in the problems. The need for inhibitory control is varied by the inclusion of distracting items appearing in some of the problems. These problems include objects that appear in both scenes, but are not the correct analogical answer. We further adapted the problem set to vary in the type of items presented in the analogies in order to examine social relational content. Social knowledge influences reasoning in cross-cultural studies involving differences in knowledge about social conduct and relationships (Cheng and Holyoak, 1985; Cosmides, 1989; Chua et al., 2005). Social abilities also affect reasoning in individuals with psychopathy (Link et al., 1977; Blair, 1995; Raine and Yang, 2006; Ermer and Kiehl, 2010). In our modified problem set half of the items contained predominantly non-living objects, whereas the remaining half contained living objects, mainly people or animals. In living problems, the relations were mainly action-based, such as chasing, kissing, and throwing, whereas non-living problems included many spatial relations, such as spatial positions, or pouring liquid. This variation in problem content was included to test whether the clinical populations show evidence of reasoning differences based on type of objects shown in the problems.

The use of a scene analogy task enabled us to test for multiple cognitive abilities important for reasoning. We predicted high performance in healthy control participants (Richland et al., 2006; Krawczyk et al., 2010; Morsanyi and Holyoak, 2010), which should enable any possible differences in performance among the clinical groups to be observed. We hypothesized that all participants would show performance reductions when problems are more relationally complex and when there are distracting items present (Markman and Gentner, 1993; Morrison et al., 2004; Richland et al., 2006). We predicted that the individuals with SZ would show deficits in analogical reasoning overall, with specific deficits in problems with distractors and in relationally complex problems due to the cognitive deficits previously associated with SZ including executive function, declarative memory, working memory and processing speed (Tamminga et al., 2010). We predicted lower performance on living compared to non-living problems in individuals with social deficits present. This includes ASD and SZ, but not healthy controls. In the ASD participants, we predicted that performance would be similar to controls on relational complexity and distraction, which would replicate the findings of Morsanyi and Holyoak (2010), who studied scene analogy in younger participants with ASD. An overall goal of the current research is to compare reasoning performance across two groups collectively known to have deficits in social cognition to determine how they are similar and how they differ.

## Materials and methods

### Participants

A total of 43 participants were enrolled in the study. The participant groups consisted of 15 individuals diagnosed with ASD, 13 individuals diagnosed with SZ, and 15 HC. Participants with SZ were recruited from University of Texas Southwestern Medical Center (UTSW). ASD and HC participants were recruited from the Dallas-Fort Worth metropolitan area. Male and female participants between the ages of 17 to 45 with a full scale IQ of at least 75 were enrolled. Six HC participants were recruited from The University of Texas at Dallas and participated for course credit. The remaining nine HC participants participated for payment. The ASD group had a current primary diagnosis of ASD as defined by the Diagnostic and Statistical Manual of Mental Disorders, 4th ed. (DSM-IV; APA, 2000) criteria (all data were collected prior to the publication of DSM-V). Our ASD sample exhibited social cognitive difficulties lacking significant language delays. For SZ participants, the diagnosis of SZ or schizoaffective disorder was supported by SCID-IV and confirmed during a consensus meeting of experienced clinicians at a SZ clinic at UTSW. This study included six individuals with Paranoid SZ, five with schizoaffective disorder and two diagnosed with SZ undifferentiated.

Participants were excluded if their clinical status (e.g., serious suicidal or homicidal risk) required inpatient or day hospital treatment, if they had a history of seizures, or if they reported substance dependence within the last 3 months or use within that last month. Participants who met all inclusion and exclusion criteria were enrolled in the study. All individuals provided written informed consent to participate in the research study. All procedures were approved by Institutional Review boards of the University of Texas at Dallas and the University of Texas at Southwestern Medical Center.

The ASD group included 11 males and 4 females with ages ranging from 17 to 34 (*M* = 21.73, *SD* = 4.39), years of education ranged from 11 to 16 (*M* = 13.26 *SD* = 1.75). The SZ group included seven males and six females with ages ranging from 18 to 39 (*M* = 30.00, *SD* = 5.72), years of education ranged from 11 to 16 (*M* = 12.92, *SD* = 2.25). All of the SZ were actively medicated and underwent a battery of neuropsychological tests. The HC group included six males and nine females with ages ranging from 18 to 45 (*M* = 23.44, *SD* = 4.00), years of education ranged from 12 to 18 (*M* = 14.22, *SD* = 2.04).

### Demographic differences

There were some differences in demographic variables, executive, and social functions among the groups (refer to Table 1). A one-way ANOVA showed a significant effect of age *F*_(2,36)_ = 10.89, *p* < 0.001 with Bonferroni-corrected *post-hoc* tests revealing that SZ were significantly older than the ASD and HC groups. There were significant differences in estimated full scale IQ using the wechsler abbreviated scale of intelligence (WASI), *F*_(2,32)_ = 31.87, *p* < 0.001. ASD estimated full scale IQ (*M* = 107.5, *SD* = 14.07), SZ (*M* = 101.67, *SD* = 12.84), and HC (*M* = 120.44, *SD* = 9.20). The HC participants displayed an estimated Full Scale Intelligence Quotient (FSIQ) above SZ and the ASD groups. FSIQ scores were not available for 10 participants (ASD = 3, SZ = 1, HC = 6).

**Table 1:** **Participant demographics, cognitive scores, and social scores**.

Participant group	Age	Education	FSIQ	Matrix reasoning	Vocabulary	Social cognition	Mind in eyes	SSQ total	SSQ friends
ASP	21.73(4.39)	13.26(1.75)	107.50(14.07)	54.57(6.25)	54.73(12.45)	46.13(11.75)	20.64(4.96)	15.50(1.78)	3.10(3.81)
SZ	30.00(5.72)	12.92(2.25)	101.67(12.84)	53.50(8.03)	48.08(10.18)	44.33(10.08)	23.67(4.05)	16.91(2.81)	3.50(5.68)
HC	23.44(4.00)	14.22(2.04)	120.44(9.20)	57.89(5.04)	64.78(6.04)	56.33(10.54)	28.33(2.73)	19.78(3.15)	11.67(7.23)

*Note. Age and education reported in years; FSIQ = Full Scale Intelligence Quotient reported as a standard score, Matrix Reasoning and Vocab = Vocabulary reported as t-scores; Social Cognition = MSCEIT score of Managing Emotions reported as t-scores; Mind in the Eyes and SSQ = Social Skills Questionnaire are reported as raw scores*.

### Vocabulary and nonverbal reasoning measures

Estimated differences in intelligence were primarily due to differences in vocabulary scores, rather than non-verbal reasoning scores. An ANOVA comparing the groups on vocabulary scores was significant *F*_(2,35)_ = 6.57, *p* = 0.004. Bonferroni-corrected *post-hoc* tests indicated that HC exhibited higher vocabulary scores (*M* = 64.78, *SD* = 6.04) than SZ (*M* = 49.25, *SD* = 8.15). We also tested the groups on matrix reasoning ability, which has previously been associated with analogical reasoning (Morsanyi and Holyoak, 2010). There were no significant group differences on this measure.

### Social function measures

We measured social perception of emotional and mental states using the Mind in the Eyes test (Baron-Cohen et al., 2001) (Mean scores are reported in Table 1). There were group differences *F*_(2,34)_ = 9.26, *p* = 0.001 with HC performing higher than ASD and SZ. A comparison of composite scores of Social Cognition reached significance *F*_(2,35)_ = 3.44, *p* = 0.04 with *post-hoc* tests revealing HC to be higher than SZ (Table 1). We used a social skills questionnaire (SSQ) that we developed to measure different aspects of social cognition including habits outside of the home and degree of social interaction. There were significant differences on the SSQ *F*_(2,30)_ = 6.42, *p* = 0.005. *Post-hoc* tests revealed that HC performed higher than ASD. The SSQ also asks a question estimating the number of friendships that involve at least monthly contact. Analysis of this measure reached significance *F*_(2,30)_ = 6.91, *p* = 0.004 with HC reporting more friendships than ASD and SZ.

### Materials

**Figure 1:**
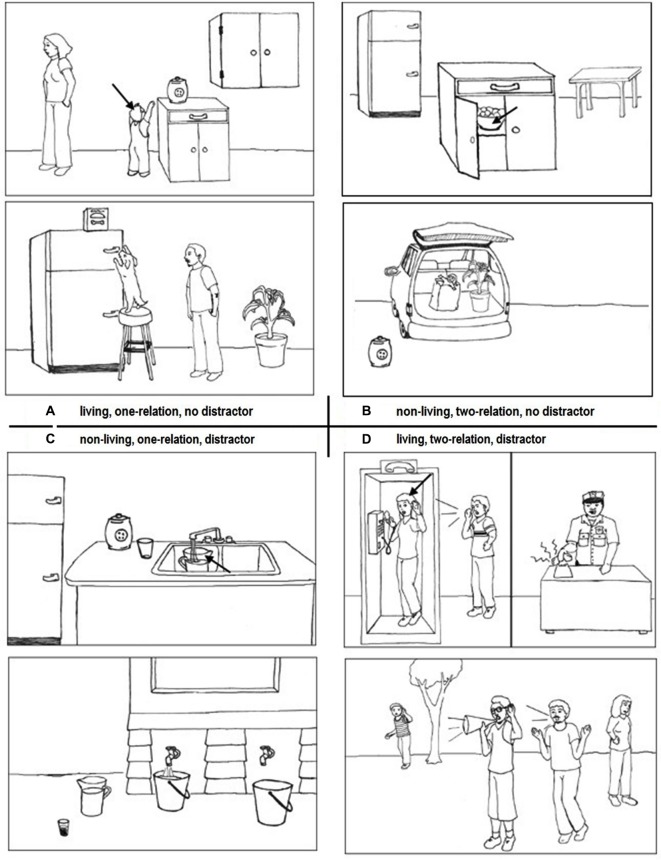
**The examples of task problem types**. The 24 problems included three factors that were varied factorially: number of relations (one-relation or two-relations), presence of distractor item (with or without distractor), and item type involved in the analogy (living or non-living items). Panel **A** shows an analogy with a single relation (reaching), involves living objects (people and a dog), and contains no distraction. Panel **B** consists of two relations (containing, and being contained), involves non-living objects (food, bowl, cabinet, groceries, bag, and car) with no distraction. Panel **C** is a one relation problem (containing) with non-living objects (water, pitcher, and bucket), with a distractor present (the pitcher in the lower picture). Panel **D** consists of two relations (receiving a message and relaying a message), living objects (people), and has a distractor (the woman in the lower picture).

We used a stimulus set of analogies developed for a prior developmental study (Richland et al., 2006) with some modifications. The analogies consisted of two pictures of scenes oriented vertically (refer to Figure 1). Each scene consisted of five items representing objects, people, or animals. Two or three of these items had relational correspondences, such as chasing, pulling, etc. An arrow pointed to a *match item* in the source scene (top picture) and this item was to be matched to a similar item in the target scene (bottom picture) in order to complete an analogy between the top and bottom scenes. The prior task (Richland et al., 2006) had been designed to investigate the effects of relational complexity and feature distraction on the reasoning abilities of children. It was controlled for knowledge of relations in regard to their youngest age group tested (approximately 3–4 years of age), therefore the relations and objects correspond to simple motion verbs (e.g., chase, fall, pull) and objects commonly encountered by children of that age group including humans, animals, and common household items.

We modified the task in two ways. First, in the original version of the task, some distractor items in the target scene were visually identical to the match item in the source scene, while other distractors appeared in a different position or orientation compared to the match item. In our modified version all distractor items were presented in a new position or orientation in the target scene. Second, we tested for the effect of category of the key items in the analogy (*living/non-living*), in addition to the original conditions employed by Richland et al. (2006), feature distraction and relational complexity, on reasoning abilities. This was accomplished by dividing the original problem set into examples in which the key items involved in the relations were alive (humans or animals) and example in which the relational items were non-living. We also added four additional problems in order to achieve an equal item numbers for counterbalancing.

The full set of 24 scene analogies was initially divided into two categories. The *living* category consisted of 12 problems in which the items relevant to the analogy were either people or animals interacting. The *non-living* category was comprised of 12 problems that included analogies in which the relevant items were non-living objects that were either manmade such as cars, trains, and lamps, or naturally occurring objects such as ponds, rocks, and trees. Within each of these sets, relational complexity was factorially-varied (one-relation or two-relation), as was the inclusion or exclusion of a distractor item in the target slide that was similar to the match item in the source slide. Figure 1 shows an example of each of the problem types. We developed four separate versions of the task. These were counterbalanced such that each of the 24 problems appeared as a one- and a two-relation problem and appeared both with and without a distractor across the four versions in order to control for specific item effects. Among the 24 items there were three items representing each possible combination.

### Design and procedure

The task was administered to participants on a laptop computer using Eprime 1.2 software.^1^ All participants were given two practice problems that demonstrated the one- and two-relation problems to familiarize them to the task. The following instructions were presented to each participant on the computer screen and read aloud by the experimenter:

You will see two pictures presented one above the other on the screen. Your job is to try and understand what is going on in each of the pictures. There will be a pattern of things happening in the top picture and your job will be to find that same pattern in the bottom picture. You will notice that one thing in the top picture of each set will have a black arrow pointing to it. Your job is to find the object in the bottom picture that best matches the one in the top picture.

Participants indicated their response by pointing to an object in the target picture presented on the computer screen. If the participant answered the sample problem correctly, the researcher would give feedback and then move on to the next sample. If the participant responded incorrectly, the researcher would repeat the description of the relationship shown in the source scene. The experimenter would then ask again which object in the target scene followed the same pattern. The participant was guided until the correct answer was reached with a correct description of the pattern. At this point, the researcher would move on to the next practice problem. If the participant was able to accurately answer the scene analogy, the researcher began the task. If the participant did not adequately perform the second example, the researcher would reiterate the instructions before moving on to the experimental task. We did not collect response time data in this experiment, as we wanted to maximize participant accuracy, comprehension of the pictures, and clarity of the item the participants chose. We had concerns that emphasizing response time would negatively influence accuracy due to the possibility of patients emphasizing speed over accuracy if this were a timed task. It is also difficult to gather accurate response times when administering the task with a finger point response.

### Statistics

Analogical reasoning data were analyzed initially with a 3 (group) × 2 (complexity) × 2 (distraction) × 2 (problem type) ANOVA. This was followed up with Bonferroni corrected *post-hoc* tests to further investigate significant main effects. To test for group differences within significant interactions we performed *post-hoc* tests with a fixed significance level of *p* < 0.01. Additionally, we ran independent 2 (distraction) × 2 (problem type) × 2 (complexity) ANOVAs on the data from each group independently to further clarify the performance observed in each of the groups. Lastly, we conducted a Pearson’s bivariate correlational analysis in order to evaluate the relationship between the analogy accuracy and each measure of social cognition.

## Results

### Combined group analysis

Results of the 3 (group) × 2 (distraction) × 2 (problem type) × 2 (complexity) ANOVA revealed three main effects on task accuracy as summarized below.

#### Participant group

**Figure 2:**
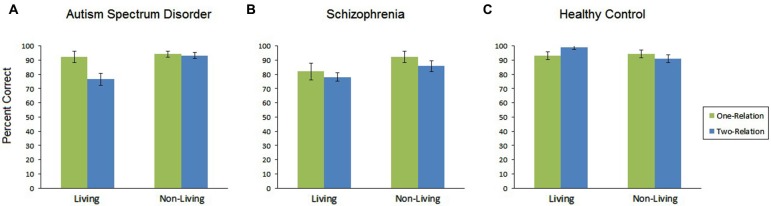
**Overall percent accuracy for each of the participant groups**. HC participants showed greater overall performance than SZ participants. There was also a main effect of problem type and a main effect of relational complexity. **(A)** ASD individuals showed a reduction in performance when solving two-relation, non-living problems. **(B)** SZ showed reductions in performance for non-living problems and a marginal reduction in performance on two-relation problems. **(C)** HC participants showed high performance across all problems types.

The ANOVA revealed a main effect of group, *F*_(1,40)_ = 5.51, *p* = 0.008. Bonferroni-corrected *post-hoc* tests indicated that the HC participants performed the task at higher accuracy levels (*M* = 94.40%) than SZ participants (*M* = 84.60%) (refer to Figure 2). No other group comparisons reached significance.

#### Relational complexity level

The number of relations had a significant effect on performance, with two-relation analogies (*M* = 86.82%) resulting in lower mean performance than one-relation analogies (*M* = 92.44%), *F*_(1,40)_ = 8.71, *p* = 0.005 (refer to Figure 2).

#### Problem type: living vs. non-living

There was a significant main effect of problem type such that problems involving living items were solved at a higher level (*M* = 93.22%) than those involving non-living items (*M* = 86.05%), *F*_(1,47)_ = 17.46, *p* < 0.001 (refer to Figure 2).

#### Presence of distractor

There was no significant effect of distractor items on overall performance (*p* > 0.05).

### Separate group analyses

#### ASD participants

The ASD group showed main effects of relation, in which one-relation problems were solved at a higher level than two-relation problems *F*_(1,14)_ = 6.18, *p* < 0.05. The ASD participants also showed a Problem Type main effect, in which living problems were solved at a higher level than non-living problems *F*_(1,14)_ = 4.68, *p* < 0.05. There was also a Relation level by Problem Type interaction *F*_(1,14)_ = 6.67, *p* < 0.05, such that two-relation living problems were solved at a higher rate than two-relation non-living problems (Figure 2A).

#### SZ participants

There was a main effect of Problem Type, such that living problems were solved more accurately than non-living problems *F*_(1,12)_ = 7.89, *p* < 0.01, as evident in Figure 2B, and a marginally significant effect of relation (one-relation more accurate than two-relation problems)* F*_(1,12)_ = 4.14, *p* = 0.065.

#### HC participants

There were no significant main effects for HC participants reflecting the overall high performance of the group. Performance was uniformly high among HC participants for each type of problem.

### Distractor analyses

The lack of an effect of distraction in the performance of all three groups was somewhat surprising. In order to further explore whether there was any tendency for the groups to miss problems when a distracting item was present, we calculated a *Distraction Score*, which was the proportion of errors made on problems with a distractor present in which participants selected the distractor item relative to errors in which participants selected any item other than the distractor (or correct relational answer). We first tested for Distractor Score proportion differences within each group comparing between Problem Type, which had been the major factor leading to errors, by using dependent-samples *t-tests*. All groups showed a tendency toward selecting more distractor items in problems with living items relative to problems with non-living items. This difference was significant for SZ participants *t*_(12)_ = 3.04, *p* < 0.01, and for HC participants *t*_(14)_ = 2.26, *p* < 0.05. Thus, non-living problems tended to elicit greater levels of distractor selection in most of the participant groups.

### Correlational analysis

We conducted a series of bivariate Pearson correlational analyses to assess the possible relationship between social cognitive measures and analogical reasoning performance. Overall accuracy on analogical reasoning was compared to our composite measure of social cognition, the Mind in the Eyes score, SSQ score, and reported number of friendships. There were significant correlations between the social cognition composite scores and analogical reasoning accuracy for all subjects combined (Pearson’s *r* = 0.36, *n* = 36, *p* = 0.031). We also observed a significant correlation between the Mind in the Eyes test and analogical reasoning accuracy (Pearson’s *r* = 0.50, *n* = 35, *p* = 0.002). These significant correlations suggest that analogical reasoning ability is related to social cognition, particularly regarding the ability to assess emotional and social motivations from eye gaze. We also tested these same correlations on each group independently. We found a highly significant correlation between analogical reasoning and Mind in the Eyes for the SZ group (Pearson’s *r* = 0.78, *n* = 12, *p* = 0.003). There were no significant correlations found between analogical reasoning and any of the social cognition measures for the HC and ASD groups.

## Discussion

Analogies become more difficult to solve when they include multiple relations among objects, including multiple relational roles played by individual objects or individuals. When perceptually similar items appear in two scenes, they tend to distract from the relational problem solving performance compared to problems that include analogous situations, which do not include perceptually or semantically similar items. These characteristics have received support from previous findings indicating that analogical correspondences are often ignored when other more basic types of similarity are present, such as perceptual or semantic similarity (Markman and Gentner, 1993; Wharton et al., 1994, 1996). Analogies also tend to be easier to form when relations are among living or animate entities, relative to situations that are relationally similar but include non-living items. In the present study HC participants performed the task with high accuracy, but when they did make errors, they tended to be on problems with greater complexity and when distractor items were present. HC participants performed the analogy task at significantly higher levels than SZ participants. ASD participants also performed the task at relatively high levels. While there was a significantly higher IQ estimate for the HC participants over the other two groups, this did not appear to be the primary reason for the differences, as ASD also showed a significantly lower estimated IQ than HC, yet showed no significant decline in analogical reasoning performance.

### Analogical reasoning in individuals with schizophrenia

The SZ participants showed reductions in overall performance relative to the HC group consistent with a prior study examining analogical reminding in SZ (Simpson and Done, 2004). The SZ group was reliably less accurate than HC on both types of problems. These results suggest a more general deficit in problem solving related to the cognitive impairments exhibited by the SZ group. The SZ group showed a trend toward reduced performance on two-relation problems, which is similar to the performance of younger age individuals (Richland et al., 2006). The SZ group also showed a clear tendency to perform better on the living problems relative to the problems that included relations among mostly non-living objects. This was a main effect across all groups, but SZ also showed this effect in the individual analysis.

The lower performance of SZ may be due to both executive and social factors. In general, the SZ performance was lower in the predicted ways based on the presence of multiple relations and distraction. Deficits in overall comprehension of the problems or deriving unique interpretations would be sufficient to lead SZ toward more distractor choices, or more errors in general as shown by their overall performance relative to HC. It is important to note that SZ individuals were not dramatically impaired on scene analogies given their overall mean accuracy. This group was taking medications and were not actively delusional, thus reasoning performance was relatively intact compared to previous reports on analogy performance that investigated delusional and non-delusional SZ participants on an analogical memory task (Simpson and Done, 2004). Also worth noting is that our SZ participants showed strong performance on the Matrix Reasoning task, which also relies heavily on relational reasoning ability, but lacks the semantic comprehension needed for solving scene analogies. The fact that our SZ participants were not impaired on the Matrix Reasoning problems suggests that they were relatively robust in executive function ability relative to other SZ patients. This suggests the possibility that greater social cognitive deficits influenced performance, as there was a strong correlation between understanding intentions and mentalizing, as measured by the Mind in the Eyes test, and analogical reasoning accuracy in SZ.

### Analogical reasoning in individuals with autism spectrum disorder

Individuals with ASD generally performed at a high level on the analogical reasoning task. This is consistent with the prior report of intact scene analogy in individuals with autism (Morsanyi and Holyoak, 2010). In that study, individuals with autism performed similarly to HC on both scene analogies and Raven’s Matrices problems. Similarly, our ASD participants exhibited high performance on our modified scene analogies task and on the Matrix Reasoning test.

One notable performance difference observed in the ASD group was that these participants tended to show more pronounced difficulties on problems that involved non-living objects compared to HC, though non-significantly (refer to Figure 2). This runs counter to our prediction that ASD would show a selective deficit on problems that involved living objects, as these individuals showed evidence of deficits in both social perception and social interactions based on the neuropsychological testing. As living problems tended to depict social interactions, we had predicted that this could lead to deficits in these problems, but this was not the case. There was an overall tendency across all groups to perform worse on the non-living problems and this is likely due to the nature of the relations among non-living items. While living objects can be depicted in action sequences, such as chasing, purposeful reaching, and calling (see Figure 1), non-living items tended to involve relations based on spatial positions or flowing of liquids. These types of relations may be less obvious and therefore lead to lower performance. Though there was not a significant interaction between problem type and distraction, it is also possible that object matches appeared more compelling when all or most items in the scenes were non-living.

### Executive functioning and reasoning

Whereas all groups performed similarly on Matrix Reasoning, there were differences in vocabulary that differentiated the SZ group from the HC group. Evidence for the role of executive function in reasoning comes from multiple sources. Two prior scene analogy experiments conducted with non-impaired populations indicated that reductions in performance followed from disruptions of working memory (through concurrent dual-tasks involving executive control) (Waltz et al., 2000), and reduction in executive control through higher anxiety (Tohill and Holyoak, 2000). Additionally, frontal lobe deficits have been shown to reduce scene analogy performance (Morrison et al., 2004), as well as tendencies toward distraction in relational reasoning (Krawczyk et al., 2008). The SZ group also exhibited a profile consistent with working memory reductions; however, the SZ group was not reliably impaired on problems that included distractor items. There is ample evidence to suggest that impairments in reasoning follow from executive function deficiencies and frontal lobe injuries, in the present study we have extended these findings to the SZ population, though our particular SZ participants were relatively intact on Matrix Reasoning problems, a relational reasoning task lacking significant semantic comprehension.

The lack of a strong linkage between Matrix Reasoning abilities and analogical reasoning in the current study has implications for theoretical perspectives on analogy. Gentner (1983) has argued that learning relational language enables reasoning by analogy (Gentner and Toupin, 1986; Goswami and Brown, 1989). This suggests that semantic memory and explicit language representations are important for analogical reasoning. Our results add to this position suggesting that relations involving human agency or interactions among living things are easier to process than relations among non-living objects. This may be due to their greater salience and disorders of social cognition such as SZ and ASD did not diminish this difference in our study. There has also been a strong link made between analogical reasoning and matrix tasks (Morsanyi and Holyoak, 2010), with both being examples of relational reasoning ability. In our study, we found both of our clinical groups to be unimpaired on Matrix Reasoning (a task lacking in semantic content), while SZ did show lower analogical reasoning performance (a task rich in semantic content), while ASD did not. This pattern of results points to the importance of semantic memory and relational knowledge in reasoning, a position advocated for in prior computational theories of analogy (Hummel and Holyoak, 1997, 2003; Morrison et al., 2004). Future work on social cognition and analogical reasoning could help to further clarify the role of social information in theories of reasoning.

### Social cognition and analogical reasoning

The results of the current study provide an initial link between social deficits and analogical reasoning performance and suggest the need for future work. The reasoning differences present in socially impaired groups to this point have primarily focused on mentalizing (Frith and Corcoran, 1996) and moral problem solving (Shenhav and Greene, 2014). There have been very few studies examining the effects of social perception deficits on reasoning. Despite significant reductions on social tasks including the Mind in the Eyes and SSQ for both ASD and SZ participants, there were no strong differences on relational responding that are directly attributable to social factors, though overall performance was related to mentalizing in SZ. Since both of our clinical groups had similar social cognitive scores, we are not able to provide clear evidence differentiating the social abilities of these two groups; however, only in SZ was mentalizing linked to lower analogical reasoning. This suggests that there are differences in social cognition between these groups, but with a relatively high functioning and limited sample, we cannot isolate the basis for these possible differences. We support the position offered by Sasson et al. (2011) provide a strong rationale for continuing to study the similarities and differences present between SZ and ASD groups.

Our results indicate that relational reasoning performance is sensitive to content with living and non-living items being relevant. Within our problem set, the social relations among living agents tended to be based on actions, such as reaching and throwing. These problems could likely have been solved on the basis of action sequence comprehension in many cases, even in individuals had difficulties perceiving or inferring the mental states of the participants involved in the problems. Future work on the role of social cognition in analogies and other forms of relational problem solving will likely benefit from manipulations of intentionality and inferences based on perceiving mental states. Without the full capacity to perform these acts of social perception, it is unlikely that relational reasoning can be carried out to its maximum degree. The correlations between analogical reasoning and social cognition, along with mentalizing support this possibility.

Ultimately more work must be done in this area to further characterize reasoning abilities in SZ and ASD. While our study is an interesting first step taking this comparative approach, we did not have a wide sample across the Autism spectrum. It is quite possible that younger individuals and those at more severe levels would present with significant deficits on analogy tasks. It is also likely that individuals with SZ may perform better on analogical reasoning if they have higher social skills and executive functioning abilities.

### Conclusions

We examined the reasoning performance of two groups that had impairments in executive and social functioning relative to healthy controls. Overall differences indicate that individuals with SZ show reductions in relational responding in analogical reasoning involving scene comprehension. Meanwhile, HC and individuals with ASD performed at high levels on the task overall. ASD did show an intriguing tendency to perform better on problems that involved living objects over those with non-living objects suggesting that they are sensitive to problem content. The clearest explanation of the deficits present in SZ links them to disorders of executive function and working memory, but in future work it will be important to examine problem content more carefully with manipulations of emotional content and mental state perception.

Overall, we have demonstrated that executive function deficits appear to have the strongest impact on relational responding, though social factors related to problem content can also have an influence. The value of this cross-diagnosis investigation is that it enables researchers to understand the relative competencies and impairments of both groups compared to unimpaired individuals. While executive functions are relevant to basic relational reasoning ability, social cognitive ability may be at least as important and future work may help to further clarify the relative similarities and differences that occur in reasoning across psychiatric and neurological populations.

## Conflict of interest statement

The authors declare that the research was conducted in the absence of any commercial or financial relationships that could be construed as a potential conflict of interest.

## Footnotes

^1^www.pstnet.com/products/e-prime
